# How Active Are Older Americans?

**Published:** 2007-06-15

**Authors:** Judy Kruger, Susan A Carlson, David Buchner

**Affiliations:** Physical Activity and Health Branch, Division of Nutrition and Physical Activity, Centers for Disease Control and Prevention; Physical Activity and Health Branch, Division of Nutrition and Physical Activity, Centers for Disease Control and Prevention, Atlanta, Ga; Physical Activity and Health Branch, Division of Nutrition and Physical Activity, Centers for Disease Control and Prevention, Atlanta, Ga

## Abstract

**Introduction:**

Regular physical activity can reduce age-related functional decline, as well people's risk for chronic diseases such as coronary heart disease, hypertension, colon cancer, and diabetes. The objective of this study was to estimate the level of participation in aerobic, muscle-strengthening, and flexibility activities among Americans aged 50 years or older.

**Methods:**

Using population-based data from the 2001 National Health Interview Survey, we classified qualified respondents (N = 11,969) according to whether they met the activity criteria used in *Healthy People 2010* goals for leisure-time participation in regular aerobic physical activity, vigorous-intensity aerobic activity, strength-training activity, and flexibility activity. We also classified respondents according to their level of aerobic activity (i.e., inactive, insufficiently active, and regularly active).

**Results:**

We estimated that 46.4% of older Americans engaged in no leisure-time aerobic activity; that 26.1% were regularly active (participated in light- to moderate-intensity aerobic activities at least 5 days per week for at least 30 minutes or vigorous-intensity activities at least 3 days per week for at least 20 minutes); that 16.2% participated in vigorous-intensity aerobic activities at least 3 days per week for at least 20 minutes; that 13.7% participated in strength-training activities at least 2 days per week; and that 24.5% participated in flexibility activities at least 1 day per week. Among the 26.1% of older Americans who were regularly active, 30.5% engaged in strengthen-training activities at least 2 days per week. Overall, only 8.2% of older Americans met the criteria for both aerobic and strength-training activity.

**Conclusion:**

As of 2001, the percentage of older Americans who met recommended activity levels of physical activity were well below the goals for U.S. adults in *Healthy People 2010*. Further efforts are needed to encourage older Americans to engage in aerobic activities and in strengthening and flexibility activities.

## Introduction

Helping people maintain their health and fitness into old age is a public health priority. Appropriately, physical activity is a leading health indicator of the *Healthy People 2010* (*HP 2010*) program (1) established by the U.S. Department of Health and Human Services (HHS). Steady increases in the number of older Americans (defined as those aged 50 years or older) (2) and in Americans' life expectancy (3) illustrate the importance of public health objectives related to the health and well-being of older adults. Physical activity is a critical part of a healthy lifestyle for older adults, and the benefits of regular physical activity are extensive. For example, regular physical activity by older adults has been shown to prevent falls and fall-related injuries (4), reduce existing functional limitations (5), and assist in reducing feelings of mild to moderate depression and anxiety (6).

This report focuses on physical activity recommendations addressed by the following five *HP 2010* objectives concerning physical activity among the overall U.S. population: reducing the prevalence of no leisure-time aerobic activity to 20% (objective 22-1), increasing the prevalence of regular moderate- or vigorous-intensity aerobic activity to 50% (objective 22-2), increasing the prevalence of vigorous-intensity aerobic activity to 30% (objective 22-3), increasing the prevalence of muscular strengthening and endurance activity to 30% (objective 22-4), and increasing the prevalence of flexibility activity to 43% (objective 22-5). (Note that objective 22-2 was modified since *HP 2010* was published in 2000: the most recent objectives can be found at http://wonder.cdc.gov/data2010/focus.htm.) We used data from the 2001 National Health Interview Survey (NHIS) (7) to evaluate how close various groups of older Americans (categorized by sex, race/ethnicity, education, and body mass index [BMI]) were to meeting these objectives in 2001.

## Methods

### Sample

The NHIS is an annual cross-sectional survey of noninstitutionalized civilian adults in the United States. It is coordinated by the National Center for Health Statistics (NCHS), and data are collected by the U.S. Bureau of the Census through face-to-face interviews. The NHIS is available in Spanish for Spanish-speaking respondents, and an interpreter is provided for respondents who need help in understanding the questions. We conducted our analysis using data from the NHIS file of 2001 NHIS participants aged 50 years or older (N = 13,060) (8). After we excluded 1091 respondents with missing information on education, any physical activity parameter, or BMI, our final study sample consisted of 11,969 adults aged 50 years or older.

### Measures

NHIS respondents were asked a set of questions related to their leisure-time physical activity. They were asked to report the frequency and duration of both light- to moderate-intensity and vigorous-intensity aerobic activities through the following questions:  "How often do you do vigorous activities for at least 10 minutes that cause heavy sweating or large increases in breathing or heart rate?" and "How often do you do light or moderate activities for at least 10 minutes that cause only light sweating or a slight to moderate increase in breathing or heart rate?" For each question, respondents reporting that they engaged in physical activity were asked to further report the duration (i.e., number of minutes or hours per activity period) of their physical activity. Those who did not report engaging in either light- to moderate-intensity or vigorous-intensity activity for at least 10 minutes at a time were categorized as inactive; those who reported engaging in light- to moderate-intensity activity for at least 30 minutes per day on at least 5 days per week or in vigorous-intensity activity for at least 20 minutes per day on at least 3 days per week were categorized as regularly active; and those who reported engaging in lesser amounts of exercise were categorized as insufficiently active.

Respondents were also asked about their participation in strengthening and flexibility activities through the following questions: "How often do you do physical activities specifically designed to strengthen your muscles, such as lifting weights or doing calisthenics?" and "How often do you do physical activities designed to stretch your muscles such as yoga, or exercises like bending side-to-side, toe touches, and leg stretches?" Respondents were categorized as meeting the criteria for strength-training activity if they reported engaging in strength training on 2 or more days per week and as meeting the criteria for flexibility activity if they reported engaging in any stretching activity at least once a week.

We calculated respondents' BMI by dividing their weight in kilograms by their height in meters squared and then divided them into three groups on the basis of their BMI: normal weight or underweight (BMI<25.0), overweight (BMI 25.0–29.9), and obese (BMI ≥30.0).

### Statistical analysis

For the descriptive analysis, we calculated overall and stratified age-adjusted estimates of physical activity prevalence within each demographic strata and used the 2000 U.S. standard population to adjust for age (8). In addition, we calculated, by aerobic activity level, the percentage of older Americans who engaged in strengthening activity at least twice a week and in flexibility activity at least once a week. We performed pairwise comparisons to calculate *t*-statistics and considered differences statistically significant at *P *<.01. We weighted the data using the final sample adult weight, which adjusts for the complex study design, nonresponses, and poststratification (by sex, age, and race/ethnicity), and we used SUDAAN (Windows version 9.0; Research Triangle Institute, Research Triangle Park, NC) for data analysis.

## Results

Overall, the study sample consisted of 46.5% men and 53.5% women; 43.8% were aged 65 years or older, 80.6% were non-Hispanic white, 22.0% were college graduates, and 24.1% had a BMI ≥30.0 (Table 1).

### No leisure-time aerobic activity

We estimated that 43.7% of older men and 48.6% of older women in the United States engaged in no leisure-time aerobic activity (Table 2) and that the prevalence of no aerobic activity was higher among those aged 65 or older than among those aged 50 to 64. By race/ethnicity, the prevalence of no leisure-time aerobic activity was higher among Hispanics (63.8%) and non-Hispanic blacks (61.6%) than among those classified as other (46.9%) or non-Hispanic white (43.0%). The prevalence of no leisure-time aerobic activity decreased with increasing levels of education and was higher among those with a BMI ≥30.0 than among those with a BMI <25.0 or among those with a BMI 25.0–29.9.

### Moderate or vigorous aerobic activity

Overall, we estimated that 26.1% of older Americans participated in light to moderate aerobic activity for at least 30 minutes per day on at least 5 days per week or in vigorous aerobic activity for at least 20 minutes per day on at least 3 days per week and that this percentage was significantly higher among men (29.0%) than women (23.7%) (Table 3). The prevalence of participation in such activity was slightly higher among those aged 50 to 64 (29.8%) than among those aged 65 or older (21.8%) and was substantially lower among Hispanics (14.7%) and non-Hispanic blacks (16.4%) than among those classified as other (25.1%) or non-Hispanic white (28.4%). By education level, the prevalence of activity was highest among college graduates (40.0%) and lowest among those with less than a high school education (14.4%). The prevalence of activity was lower among those with a BMI ≥30 than among those in the other two BMI categories.

### Vigorous aerobic activity

Only 16.2% of older Americans met the vigorous activity criterion (i.e., at least 20 minutes per day on at least 3 days per week), and the demographic patterns in the percentage doing so were similar to those for light/moderate or vigorous activity (Table 4). The prevalence of vigorous activity was higher among men (19.3%) than among women (13.4%); higher among those aged 50 to 64 (20.4%) than among those aged 65 or older (11.1%); lower among Hispanics (8.3%) and non-Hispanic blacks (8.8%) than among non-Hispanic whites (17.9%); higher among college graduates (28.3%) than among those with less education; and lower among those with a BMI ≥30.0 than among those in the other two BMI categories.

### Strength-training activity

Only 13.7% of older Americans participated in strength training at least twice a week (Table 5). Once again, the percentage was higher among men (15.3%) than among women (12.4%); higher among those aged 50 to 64 (16.3%) than those aged 65 or older (10.7%); higher among those classified as other race/ethnicity (16.6%) and among non-Hispanic whites (15.0%) than among non-Hispanic blacks (8.3%) or Hispanics (6.0%); highest among college graduates (24.2%) and lowest among those with less than a high school education (5.7%); and higher among those with a BMI <25.0 than among those with a BMI ≥30.0.

### Flexibility activity

Only 24.5% of older Americans participated in flexibility activity at least once a week (Table 6). Unlike the other activity categories, the age-adjusted prevalence was higher among women (26.2%) than men (22.7%); however, like the other categories, the prevalence was lower among those aged 65 or older than among those aged 50 to 64; lower among Hispanics (15.9%) than among all other racial/ethnic groups; higher among college graduates (35.4%) than among those with less education; and lower among those with a BMI ≥30.0 than among those in the other two BMI categories.

We also observed a positive relationship between participation in leisure-time aerobic activity and participation in strength training. Of those who were regularly active, we estimated that 30.5% also met the strength-training criterion, whereas among those who were inactive, we estimated that only 3.2% did so (Table 7). Similarly, among those who were regularly active, 42.6% engaged in flexibility activity at least once a week, compared with only 9.5% among those who were inactive. Overall, we estimated that only 8.2% (95% CI, 7.6–8.8) of older Americans were both regularly active and engaged in strengthening activity at least twice a week and that only 6.3% (95% CI, 5.8–6.9) participated in recommended amounts of leisure-time aerobic, strength-building, and flexibility exercises (data not shown).

## Discussion

Our results showed that, as of 2001, older Americans did not meet any of the five *HP 2010* physical activity objectives for the general adult U.S. population (22-1 through 22-5). Although some subgroups were closer than others to reaching *HP 2010* objective 22-2 (50% participation in regular leisure-time aerobic activity), the prevalence of regular aerobic activity among older Americans was generally low. We also found that the prevalence of strength training was substantially lower than the *HP 2010* target of 30% and that only 8.2% of older Americans engaged in recommended levels of both regular aerobic activities and muscle-strengthening activities. These findings may aid efforts to meet national physical activity objectives by providing baseline estimates.

Other national surveys have shown that participation in physical activity decreases with age. For example, unadjusted findings from *Healthy People DATA2010* indicate that from 1997 to 2004, Americans' rate of participation in all types of physical activity decreased with increasing age (9). Age-adjusted findings from the 2001 Behavioral Risk Factor Surveillance System (BRFSS) survey also showed that levels of both moderate and vigorous activity decreased with increasing age (10). A number of barriers can restrict physical activity as people age, including functional limitations (11), lack of access to activity programs (12), decreased confidence in one's ability to engage in physical activity (13), lack of knowledge about the benefits of physical activity (14), and not viewing exercise as an appropriate activity for one's age (15). Lack of physical activity among community-dwelling older adults has been associated with an increased risk for a decline in functional status (16), and physical activity has been shown to be health-enhancing even among those with functional limitations (11). One way to encourage older adults to become more physically activity is to increase their access to locations that offer physical activity and recreation opportunities. Settings that older adults regularly visit (such as senior centers, community centers, and recreational facilities) may have staff on site who could provide the encouragement and equipment necessary to help older adults incorporate physical activity into their daily lives.

The demographic patterns we found in rates of participation in various physical activities are not unique to Americans aged 50 or older: similar demographic patterns have been observed across the age spectrum (9). In addition, our results confirm previous findings that, as a group, older adults have a higher prevalence of no leisure-time aerobic activity than younger adults (17) and that the age-related difference is particularly high among those with less than a high school education (18,19). Predictably, we found that participation rates in all categories of activities that we examined were highest among those who had a college education. We also found substantial racial/ethnic differences in the prevalence of physical activity. Our finding that Hispanics were generally less likely to engage in leisure-time physical activity than were non-Hispanic whites is consistent with research findings in the general adult population (20–22). To eliminate this disparity between Hispanics and non-Hispanic whites, Poston and colleagues suggested that culturally appropriate interventions to increase physical activity among Hispanics need more than bilingual facilitators and materials (23). Analyses of activity levels by demographic subgroups (such as we did in this study) may be helpful in providing direction for additional research or in identifying appropriate target populations for interventions.

Our findings showed that only 8.2% of older Americans were engaging in recommended levels of both aerobic and muscle-strengthening activities. Strong evidence suggests that physical activity, especially activities that focus on muscle strengthening, can help older adults enjoy daily activities, reduce their risk of falling, and maintain an independent living status (24). A 2006 publication of the U.S. Department of Health and Human Services, *A Healthier You,* suggests that adults, regardless of age, can improve their overall health by engaging both in moderate- or vigorous-intensity aerobic activity and in strength training (25).

The findings of this analysis are subject to several limitations, including possible misclassification errors caused by survey participants overestimating their level of physical activity or by their possible confusion in being asked about their participation in vigorous-intensity activity before being asked about their participation in light- or moderate-intensity activity. Physical activity levels can also be tracked by using a number of commercial instruments that measure performance, such as changes in heart rate, time engaged in physical activity, or number of steps taken. Although these instruments can be used to validate self-reported physical activity and are the preferred gold standard in any study, their use is often not feasible or cost-effective in large population-based studies. However, the NHIS questions are similar to questions used in BRFSS surveys, which have been shown to have acceptable levels of validity (26,27). Other limitations of this study include limited opportunities for NHIS respondents to provide details about specific activities they engaged in (e.g., walking, running, or dancing for aerobic endurance activity), about their perceptions of barriers to and incentives for physical activity, or about where they performed any reported physical activity (e.g., community centers, fitness centers, senior centers); lack of information about the possible effect of language differences on the accuracy of people's reported behavior; and the study's reliance on data for only one year (2001).

The strengths of this study include its use of data drawn from a national survey of U.S. households whose questions allowed us to compare various physical activity rates among older Americans in 2001 with *HP 2010* objectives. Our findings, which show that the proportion of sedentary older Americans remains high, can help define target populations for future physical activity interventions and may assist health education efforts to make older Americans more aware of the benefits of regular physical activity, including its potential to delay the progressive decline associated with aging. Only through such targeted promotion of physical activity can the national goals in *HP 2010* be reached.

## Figures and Tables

**Table 1:** Estimated Distribution^a^ of Select Characteristics Among U.S. Adults Aged 50 Years or Older, Overall and by Sex, 2001 National Health Interview Survey (N = 11,969)

Characteristic	Total% (95% CI)	Men% (95% CI)	Women% (95% CI)
**Age group, y**			
50–64	56.2 (55.1–57.2)	59.3 (57.6–60.9)	53.5 (52.1–54.9)
≥65	43.8 (42.8–44.9)	40.7 (39.1–42.4)	46.5 (45.1–47.9)
**Race/ethnicity**			
Non-Hispanic white	80.6 (79.5–81.5)	81.5 (80.2–82.8)	79.7 (78.4–80.9)
Non-Hispanic black	9.3 (8.6–10.0)	8.4 (7.6–9.2)	10.1 (9.2–11.1)
Hispanic	7.0 (6.6–7.8)	7.0 (6.2–7.9)	7.3 (6.6–8.2)
Other^b^	3.0 (2.6–3.5)	3.1 (2.6–3.8)	2.9 (2.4–3.5)
**Education**			
<High school graduate	23.2 (22.3–24.1)	22.5 (21.2–23.8)	23.8 (22.6–25.0)
High school graduate	31.7 (30.8–32.7)	27.9 (26.5–29.3)	35.0 (33.8–36.4)
Some college	23.1 (22.1–24.0)	22.6 (21.3–24.0)	23.5 (22.3–24.8)
College graduate	22.0 (21.0–23.0)	27.0 (25.6–28.5)	17.6 (16.6–18.8)
**Body mass index (kg/m^2^)**			
Normal (<25.0)	36.8 (35.7–37.8)	30.3 (28.8–31.8)	42.5 (41.0–44.0)
Overweight (25.0–29.9)	39.1 (38.1–40.1)	46.0 (44.4–47.6)	33.0 (31.8–34.3)
Obese (≥30.0)	24.1 (23.3–25.1)	23.7 (22.4–25.0)	24.5 (23.3–25.7)
**All U.S. Adults Aged 50 or Older**	**100.0 (NA)**	**46.5 (45.5–47.6)**	**53.5 (52.4–54.5)**

CI indicates confidence interval; NA, not applicable.

a Percentage estimates (except for those by age group) were weighted and age-adjusted to the 2000 U.S. standard population for the age groups in the table.

b Other refers to non-Hispanic American Indian, Alaskan Native, or Asian/Pacific Islander.

**Table 2:** Estimated Percentage^a^ of Americans Aged 50 Years or Older Who Engaged in No Leisure-Time Aerobic Activity,^b^ by Select Characteristics, 2001 National Health Interview Survey (N = 11,969)

Characteristic	Total% (95% CI)	Men% (95% CI)	Women% (95% CI)
**Age group, y **			
50–64	41.2 (39.7–42.6)	40.5 (38.4–42.6)	41.8 (39.9–43.9)
≥65	52.6 (51.0–54.2)	47.4 (45.1–49.8)	56.6 (54.6–58.6)
**Race/ethnicity**			
Non-Hispanic white	43.0 (41.7–44.4)	40.0 (38.1–41.9)	45.4 (43.7–47.2)
Non-Hispanic black	61.6 (58.5–64.6)	58.8 (54.2–63.3)	63.5 (60.0–66.9)
Hispanic	63.8 (60.8–66.7)	66.9 (61.9–71.6)	61.1 (57.1–64.9)
Other^c^	46.9 (39.3–54.7)	43.8 (33.0–55.3)	49.3 (40.0–58.7)
**Education**			
<High school graduate	65.4 (63.2–67.5)	64.5 (61.2–67.6)	65.9 (62.9–68.8)
High school graduate	49.7 (47.8–51.7)	49.2 (46.1–52.4)	49.8 (47.5–52.1)
Some college	41.7 (39.5–43.9)	40.9 (37.5–44.3)	42.2 (39.3–45.2)
College graduate	27.1 (24.9–29.5)	24.1 (21.3–27.1)	31.3 (28.3–34.6)
**Body mass index (kg/m^2^)**			
Normal (<25.0)	44.6 (43.0–46.2)	44.6 (41.8–47.4)	44.5 (42.6–46.5)
Overweight (25.0–29.9)	43.8 (42.0–45.6)	40.8 (38.4–43.2)	47.3 (44.7–49.8)
Obese (≥30.0)	52.7 (50.5–54.9)	47.3 (44.1–50.6)	56.9 (54.2–59.6)
**All U.S. adults aged 50 or older**	**46.4 (45.3–47.6)**	**43.7 (42.0–45.3)**	**48.6 (47.1–50.0)**

CI indicates confidence interval.

a Percentage estimates (except those by age group) were age-adjusted to the 2000 U.S. standard population for the age groups in the table.

b Survey respondents who did not report engaging in any leisure-time physical activity were considered to have engaged in no leisure-time physical activity.

c Other refers to non-Hispanic American Indian, Alaskan Native, or Asian/Pacific Islander.

**Table 3:** Estimated Percentage^a^ of Americans Aged 50 Years or Older Who Engaged in Regular Aerobic Activity,^b^ by Select Characteristics, 2001 National Health Interview Survey (N = 11,969)

Characteristic	Total% (95% CI)	Men% (95% CI)	Women% (95% CI)
**Age group, y **			
50–64	29.8 (28.4–31.2)	32.0 (30.0–34.0)	27.6 (25.8–29.5)
≥65	21.8 (20.6–23.2)	25.5 (23.5–27.7)	19.0 (17.4–20.7)
**Race/ethnicity**			
Non-Hispanic white	28.4 (27.3–29.6)	31.3 (29.6–33.1)	25.9 (24.5–27.5)
Non-Hispanic black	16.4 (14.3–18.7)	20.1 (16.1–24.8)	13.7 (11.0–17.0)
Hispanic	14.7 (12.6–17.1)	15.4 (12.0–19.6)	14.2 (11.6–17.3)
Other^c^	25.1 (19.8–31.2)	25.5 (17.3–35.8)	24.1 (17.9–31.6)
**Education**			
<High school graduate	14.4 (12.9–16.0)	14.9 (12.8–17.3)	14.3 (12.2–16.6)
High school graduate	22.0 (20.4–23.6)	24.4 (22.0–26.9)	20.3 (18.4–22.5)
Some college	29.7 (27.5–31.9)	32.2 (29.0–35.5)	27.7 (25.1–30.4)
College graduate	40.0 (37.6–42.6)	41.4 (38.2–44.7)	38.2 (34.8–41.6)
**Body mass index (kg/m^2^)**			
Normal (<25.0)	29.2 (27.7–30.9)	30.2 (27.7–32.9)	28.7 (26.8–30.6)
Overweight (25.0–29.9)	27.4 (25.9–28.9)	30.7 (28.6–32.8)	23.3 (21.3–25.5)
Obese (≥30.0)	20.2 (18.4–22.2)	25.4 (22.6–28.6)	16.1 (14.1–18.4)
**All U.S. adults aged 50 or older**	**26.1 (25.2–27.2)**	**29.0 (27.6–30.5)**	**23.7 (22.5–25.0)**

CI indicates confidence interval.

a Percentage estimates (except for those by age group) were age-adjusted to the 2000 U.S. standard population for the age groups in the table.

b Regular activity refers to light- or moderate-intensity aerobic activity for at least 30 minutes ≥5 times/week or vigorous-intensity activity for at least 20 minutes, ≥3 times/week.

c Other refers to non-Hispanic American Indian, Alaskan Native, or Asian/Pacific Islander.

**Table 4:** Estimated Percentage^a^ of Americans Aged 50 Years or Older Who Engaged in Regular Vigorous-Intensity Aerobic Activity for at Least 20 minutes on 3 or More Days per Week,^b^  by Select Characteristics, 2001 National Health Interview Survey (N = 11,969)

Characteristic	Total% (95% CI)	Men% (95% CI)	Women% (95% CI)
**Age group, y **			
50–64	20.4 (19.2–21.7)	23.3 (21.5–25.2)	17.6 (16.1–19.3)
≥65	11.1 (10.2–12.0)	14.5 (12.9–16.2)	8.5 (7.5–9.6)
**Race/ethnicity**			
Non-Hispanic white	17.9 (17.0–18.8)	21.2 (19.8–22.8)	14.9 (13.8–16.1)
Non-Hispanic black	8.8 (7.4–10.5)	11.5 (8.8–14.8)	6.8 (4.9–9.5)
Hispanic	8.3 (6.8–10.1)	8.9 (6.4–12.2)	8.0 (6.1–10.5)
Other^b^	13.4 (9.5–18.5)	13.9 (8.9–21.1)	12.4 (7.4–19.8)
**Education**			
<High school graduate	7.3 (6.2–8.6)	8.6 (6.8–10.8)	6.3 (5.0–8.0)
High school graduate	11.9 (10.8–13.2)	14.8 (12.8–17.0)	10.0 (8.6–11.6)
Some college	18.2 (16.5–20.0)	19.9 (17.2–22.9)	16.8 (14.7–19.1)
College graduate	28.3 (26.2–30.5)	31.2 (28.3–34.3)	24.3 (21.5–27.3)
**Body mass index (kg/m^2^)**			
Normal (<25.0)	18.1 (16.9–19.5)	19.1 (16.9–21.4)	17.6 (16.0–19.3)
Overweight (25.0–29.9)	17.2 (16.0–18.5)	20.8 (19.0–22.6)	12.7 (11.2-14.4)
Obese (≥30.0)	12.2 (10.8–13.8)	17.5 (15.1–20.3)	7.8 (6.4–9.4)
**All U.S. adults aged 50 or older**	**16.2 (15.4–17.0)**	**19.3 (18.0–20.6)**	**13.4 (12.5–14.4)**

CI indicates confidence interval.

a Percentage estimates (except for those by age group) were age-adjusted to the 2000 U.S. standard population for the age groups in the table.

b Other refers to non-Hispanic American Indian, Alaskan Native, or Asian/Pacific Islander.

**Table 5:** Estimated Percentage^a^ of Americans Aged 50 Years or Older Who Engaged in Strength Training Activity at Least Twice a Week, by Select Characteristics, 2001 National Health Interview Survey (N = 11,969)

Characteristic	Total% (95% CI)	Men% (95% CI)	Women% (95% CI)
**Age group, y**			
50–64	16.3 (15.2–17.4)	17.5 (16.0–19.1)	15.1 (13.6–16.7)
≥65	10.7 (9.7–11.7)	12.6 (11.1–14.3)	9.2 (8.1–10.4)
**Race/ethnicity**			
Non-Hispanic white	15.0 (14.1–15.9)	16.5 (15.2–17.9)	13.7 (12.5–14.9)
Non-Hispanic black	8.3 (6.8–10.2)	9.3 (7.0–12.4)	7.6 (6.0–9.6)
Hispanic	6.0 (4.7–7.7)	7.6 (5.4–10.6)	4.8 (3.5–6.7)
Other^b^	16.6 (12.0–22.5)	16.9 (9.8–27.7)	16.2 (11.7–24.0)
**Education**			
<High school graduate	5.7 (4.7–6.8)	6.3 (4.8–8.3)	5.1 (4.0–6.5)
High school graduate	9.5 (8.3–10.8)	10.1 (8.3–12.1)	9.2 (7.8–10.9)
Some college	16.4 (14.8–18.1)	17.3 (14.8–20.0)	15.8 (13.8–18.2)
College graduate	24.2 (22.3–26.3)	25.7 (22.9–28.6)	22.2 (19.7–25.0)
**Body mass index (kg/m^2^)**			
Normal (<25.0)	16.8 (15.5–18.2)	17.1 (15.0–19.4)	16.7 (15.0–18.4)
Overweight (25.0–29.9)	14.1 (13.0–15.4)	16.5 (14.8–18.4)	11.2 (9.8–12.8)
Obese (≥30.0)	8.8 (7.8–10.0)	10.7 (8.8–12.8)	7.2 (6.0–8.8)
**All U.S. adults aged 50 or older**	**13.7 (13.0–14.5)**	**15.3 (14.1–16.5)**	**12.4 (11.4–13.4)**

CI indicates confidence interval.

a Percentage estimates (except for those by age group) were age-adjusted to the 2000 U.S. standard population for the age groups in the table.

b Other refers to non-Hispanic American Indian, Alaskan Native, or Asian/Pacific Islander.

**Table 6:** Estimated Percentage^a^ of Americans Aged 50 Years or Older Who Engaged in Flexibility Activity at Least Once a Week,^a^ by Select Characteristics, 2001 National Health Interview Survey (N = 11,969)

Characteristic	Total% (95% CI)	Men% (95% CI)	Women% (95% CI)
**Age group, y**			
50–64	27.7 (26.5–29.0)	25.3 (23.5–27.2)	30.1 (28.2–32.0)
≥65	20.8 (19.5–22.1)	19.6 (17.7–21.7)	21.7 (20.1–23.4)
**Race/ethnicity**			
Non-Hispanic white	25.5 (24.4–26.6)	23.7 (22.2–25.3)	27.2 (25.6–28.7)
Non-Hispanic black	21.4 (18.4–24.8)	18.3 (14.5–22.7)	23.8 (20.1–28.0)
Hispanic	15.9 (13.9–18.1)	13.3 (10.7-16.4)	18.0 (15.3–21.1)
Other^b^	31.8 (25.2–39.1)	30.6 (21.3–41.7)	32.4 (24.4–41.6)
**Education**			
<High school graduate	14.5 (13.1–16.1)	12.7 (10.8–15.0)	16.1 (14.2–18.1)
High school graduate	20.2 (18.6–21.8)	17.4 (15.3–19.7)	22.2 (20.2–24.3)
Some college	28.6 (26.6–30.7)	23.7 (20.9–26.7)	32.8 (30.3–35.5)
College graduate	35.4 (33.1–37.7)	34.1 (31.1–37.3)	37.1 (33.6–40.8)
**Body mass index (kg/m^2^)**			
Normal (<25.0)	28.1 (26.5–29.7)	25.6 (23.1–28.2)	29.6 (27.6–31.7)
Overweight (25.0–29.9)	25.2 (23.7–26.6)	24.1 (22.2–26.1)	26.4 (24.4–28.6)
Obese (≥30.0)	18.6 (17.0–20.2)	16.5 (14.4–18.9)	20.4 (18.2–22.8)
**All U.S. adults aged 50 or older**	**24.5 (23.6–25.5)**	**22.7 (21.3–24.1)**	**26.2 (24.9–27.6)**

CI indicates confidence interval.

a Percentage estimates (except for those by age group) were age-adjusted to the 2000 U.S. standard population for the age groups found in the table.

b Other refers to non-Hispanic American Indian, Alaskan Native, or Asian/Pacific Islander.

**Table 7:** Estimated Percentage^a^ of Americans Aged 50 Years or Older Who Participated in Strengthening and Flexibility Activities, by Aerobic Activity Level, 2001 National Health Interview Survey (N = 11,969)

**Activity**	**Regularly active^b^ % (95% CI)**	**Insufficiently active^c^ % (95% CI)**	**Inactive^d^ % (95% CI)**
Strengthening ≥2 days/week	30.5 (28.8–32.3)	14.5 (13.0–16.0)	3.2 (2.7–3.9)
Flexibility ≥1 day/week	42.6 (40.6–44.6)	31.9 (30.0–33.8)	9.5 (8.5–10.5)

CI indicates confidence interval.

a Percentage estimates were age-adjusted to the 2000 U.S. standard population for 2 age groups: 50–64 years and ≥65 years.

b Engaged in light- to moderate-intensity aerobic activity for at least 30 minutes ≥5 times/week or vigorous-intensity aerobic activity for at least 20 minutes ≥3 times/week.

c Engaged in some light- to moderate- and/or vigorous-intensity aerobic activity, but at a lower frequency or duration than the minimum for regularly active.

d Engaged in no leisure-time aerobic activity.
